# Development of a Wireless Telemetry Sensor Device to Measure Load and Deformation in Orthopaedic Applications

**DOI:** 10.3390/s20236772

**Published:** 2020-11-27

**Authors:** William D. Anderson, Sydney L. M. Wilson, David W. Holdsworth

**Affiliations:** 1 School of Biomedical Engineering, Western University, London, ON N6A 3K7, Canada; wander2@uwo.ca (W.D.A.); swils226@uwo.ca (S.L.M.W.); 2 Robarts Research Institute, Western University, London, ON N6A 5K8, Canada; 3 Department of Medical Biophysics, Western University, London, ON N6A 5C1, Canada; 4 Department of Surgery, Western University, London, ON N6A 4V2, Canada

**Keywords:** accelerometer, capacitive transducers, deformation, load sensor, orthopaedic implants, pressure sensors, strain, telemetry, wireless

## Abstract

Due to sensor size and supporting circuitry, in-vivo load and deformation measurements are currently restricted to applications within larger orthopaedic implants. The objective of this study is to repurpose a commercially available low-power, miniature, wireless, telemetric, tire-pressure sensor (FXTH87) to measure load and deformation for future use in orthopaedic and biomedical applications. The capacitive transducer membrane was modified, and compressive deformation was applied to the transducer to determine the sensor signal value and the internal resistive force. The sensor package was embedded within a deformable enclosure to illustrate potential applications of the sensor for monitoring load. To reach the maximum output signal value, sensors required compressive deformation of 350 ± 24 µm. The output signal value of the sensor was an effective predictor of the applied load on a calibrated plastic strain member, over a range of 35 N. The FXTH87 sensor can effectively sense and transmit load-induced deformations. The sensor does not have a limit on loads it can measure, as long as deformation resulting from the applied load does not exceed 350 µm. The proposed device presents a sensitive and precise means to monitor deformation and load within small-scale, deformable enclosures.

## 1. Introduction

Instrumentation of sensor packages within orthopaedic implants has long been a challenge due to requirements related to the size of the sensor package, the need for wireless telemetry, and low-power consumption [1,2]. Embedded sensors can be tasked to measure load, strain, temperature, and acceleration. These variables can allow scientists and clinicians to diagnose and monitor implant wear, implant migration, tissue infection, and other factors such as bone healing. In-vivo implant data aids in improving orthopaedic implant design and patient rehabilitation practices. Although numerous instrumented implants embedded with sensors have been developed to measure load and strain, deficiencies remain. Most sensor packages are too large to be incorporated into smaller orthopaedic components, such as fracture fixation plates, intervertebral spinal fusion cages, and high tibial osteotomy implants, to name a few. These packages are not limited by the size of the sensor itself, but by size of accompanying signal processing, wireless telemetry, and power-management aspects. There is a clear need for a miniature sensor package with integrated power management and radio frequency (RF) transmission components that is capable of measuring load and strain inside smaller orthopaedic implants.

Much work has been done by others to develop wireless telemetric sensor packages capable of measuring and transmitting in-vivo load, strain, displacement, temperature, and acceleration. Telemetric sensor packages have been embedded within larger orthopaedic components such as shoulder [3], hip [4,5,6,7,8,9,10], knee replacements [11,12,13,14,15,16], external fixation devices [17,18], and spinal implants [19,20]. Previous work with embedded sensors in internal fixation devices has been unsuccessful in intramedullary nails [21,22]. Sensor packages have been feasible in fracture fixation plates [23,24,25,26,27]; however, the sensorized implants tend to be bulky without featuring supporting sensors and functionalities associated with integrated microcontroller units. For example, supporting sensors for variables such as acceleration, temperature, pH, and biomarkers are not essential for direct load and displacement measurements, but can provide useful additional information about the environment surrounding the implant.

Most instrumented implants utilize a 9-channel telemetry system for in-vivo load measurements developed by Graichen et al. [28]. To make load and strain measurements, this system features multiple semiconductor strain gauges connected to a transmitter circuit that sends data through an RF transmitter. The 9-channel telemetry package is very effective at obtaining accurate load measurements over a long period of time. The disadvantage of this telemetry sensor package is that it is too large to be incorporated within smaller orthopaedic components, but it has been shown to be feasible in shoulder, hip, knee, and vertebral body replacements. The 9-channel telemetry package occupies a large volume within the orthopaedic components. In comparison, the device proposed in this study is significantly smaller in size and may only occupy a small internal region of the implant, but it does not have multiple channels dedicated to load measurements. In larger components, it may be advantageous to use the 9-channel system with increased measurement capability. However, in smaller orthopaedic components, such as spinal fusion cages, the proposed device would be advantageous as it is feasible for use in measuring a single compressive load.

Many previously described embedded sensors have used inductive coupling as a power source, as it eliminates the need to periodically replace a battery; however, this method is limited as to when and where measurements can be taken, due to the lack of an internal power supply. To make measurements with such a device, patients must strap a metallic coil around the external surface of the body in the region where the sensor package is located. Additionally, the external coil needs to be periodically recharged or wired to a power supply. In cases where continuous monitoring is necessary, alternative methods of power management are required. Internal battery sources are rarely used due to their limited lifespan and the risk of chemical exposure, however, they are beneficial in applications where measurements are taken over shorter time periods, such as monitoring implant strain during bone healing. A piezoresistive energy harvesting system that powers an orthopaedic implant through body movement would be the ideal solution as it allows for long-term continuous monitoring and data transfer. Energy harvesting has been proven to be successful in total knee replacements [29]. Alternatively, energy harvesting from human kinetics remains a challenge in other orthopaedic implants, as it is not capable of generating sufficient levels of current necessary for data acquisition, storage, and transmission in most low-power telemetry sensor packages [30,31,32].

Another approach, similar to the 9-channel telemetry system, was developed by D’Lima et al. [11] and Kaufman et al. [14] to measure intra-articular tibial loads. This method also features strain gauges placed in strategic locations around the tibial tray to determine the force on each load cell and the center of pressure. The load cells connect to signal conditioning equipment, an analog to digital converter (ADC), and a micro-RF transmitter. The tibial package, although successful, still faces similar size and power management restrictions as the previously described 9-channel telemetry system. 

A telemetric intramedullary nail to monitor fracture healing was developed in [21]. A printed circuit board (PCB) and semiconductor strain gauge were used to measure anterior-posterior nail strain and axial compression forces in a small orthopaedic component. However, this method of measuring load and strain was not feasible for implementation within an intramedullary nail as this package was not able to effectively monitor load changes. The common theme of successful sensor packages is the method of load and strain transduction, inductive coupling power supply, and the size of orthopaedic implants they are embedded within. These packages all feature an array of strain gauges connected to signal processing equipment and an RF transmitter and are situated inside larger orthopaedic components. For embedded sensing devices to be successful in smaller orthopaedic components, an alternative mechanism of load and strain transduction must be developed.

**Figure 1:**
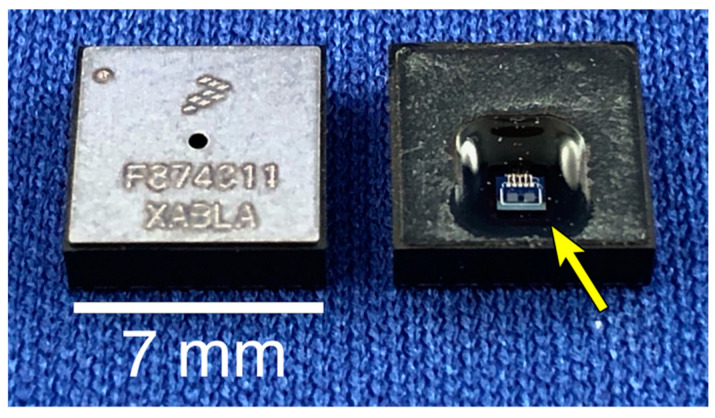
FXTH87 tire pressure sensor component. The intact package, as provided by the manufacturer is shown on the left. On the right, the protective cover has been removed, exposing the capacitive transducer (arrow).

Recent advancements in the field of automotive sensor design, specifically microelectromechanical system (MEMS)-based technology, have made alternative methods of load and strain transduction feasible for implantation within orthopaedic components. The FXTH87 (Figure 1) is a commercially available tire-pressure sensor package developed by NXP Semiconductors (Eindhoven, The Netherlands). This wireless package includes a pressure sensor, a two-axis accelerometer, and a temperature sensor. With the modifications proposed in this report, this package is ideally suited for future use in orthopaedic applications as it can be incorporated inside small enclosures and has the necessary components to record and transmit sensor data. 

This paper proposes technical modifications to a commercially available wireless telemetric tire-pressure sensor that converts it into a compact, low-power, load and displacement sensor that may be feasible for future use in orthopaedic applications. The full range of physical deformation of the capacitive transducer was quantified. A relationship was reported between compressive deformation and output signal value from the sensor package. In addition, the sensor package was calibrated to measure the load required to cause the compressive deformation. In the event that the sensor package would be used to track activity or to activate sensor transmissions, the two-axis accelerometer was calibrated over a range of ±5 *g*. The exact power requirements and RF transmission distances were obtained in order to estimate its feasibility for future use in an orthopaedic implant. To illustrate the feasibility of our package, the modified sensor was calibrated when embedded inside a small-scale custom strain member that was designed to transduce compression. The resulting correlation between deformation and signal, accompanied with its size and functionality, make this device an effective solution for measuring load, displacement, acceleration, and temperature. This approach may be applicable for use in many small orthopaedic implants, including fracture fixation plates, intervertebral spinal fusion cages, and high tibial osteotomy implants.

## 2. Materials and Methods

### 2.1. FXTH87 Sensor Characteristics

Due to the unique environment of orthopaedic sensing applications, the ideal sensor would feature a low-power, stand-alone, miniature sensor package with a novel approach to measuring load and strain. The proposed sensor package, the FXTH870511DT1 (FXTH87), features an 8-bit 4 MHz CPU, 315/434 MHz RF transmitter, 6 channel 10-bit ADC, 125 kHz LF receiver, pressure sensor, dual-axis accelerometer, temperature sensor, and additional components, making the FXTH87 a fully functional wireless telemetry package [33]. All sensors, transmitting equipment, memory, and CPU for the FXTH87 are housed inside a miniature package measuring 7 mm × 7 mm × 2 mm, making it one of the smallest pressure sensing packages in the world. This package utilizes a capacitive pressure transducer to obtain pressure measurements. Capacitive pressure transducers feature two metallic plates held in parallel and separated by a dielectric medium [34,35]. The bottom plate, known as the electrode, is held fixed and used as a reference for the top plate. The top plate is called the diaphragm and it can move closer to the electrode. As the diaphragm moves toward the electrode and the distance between the two plates decreases, the output capacitance increases. In its commercial configuration, the capacitive pressure transducer is calibrated to measure changes in air pressure. For the sensor configuration described in this study, instead of measuring air pressure, the onboard capacitive transducer was modified (as described below) and then calibrated to measure load and deformation by manipulating the distance between the electrode and the diaphragm using a 3D printed mechanical indenter.

### 2.2. FXTH87 Circuit Board Design

**Figure 2:**
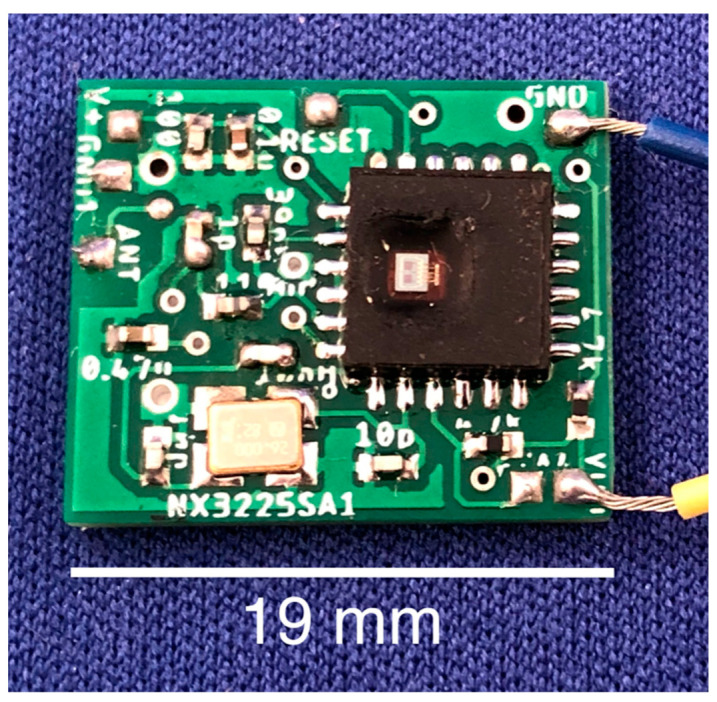
FXTH87 tire pressure sensor mounted to custom PCB without LF capabilities.

A PCB for the main sensor chip and surrounding circuitry was designed in EAGLE Autodesk (Autodesk, San Rafael, CA, USA) following basic principles for optimal RF transmission [36]. Two different PCB’s were designed. The first board was only capable of transmitting RF signals and was 19 mm × 16 mm × 0.4 mm (excluding circuit components). The second board acted as a transceiver which could send RF signals and receive LF signals; the received LF signals act to control the functionality and programming of the FXTH87. The additional functionality came at the cost of a slightly larger size: 26 mm × 16 mm × 0.4 mm. For the purpose of the experiments in this paper, the LF functionality was not required and the one-way transmitter PCB was used (Figure 2). 

It is important to note that a dedicated antenna component was not required in this design. RF communication was achieved by transmitting from a copper pad (1.1 × 1.1 mm) which is labeled as ANT on the left side of the PCB (Figure 2). Given that the FXTH87 is a quad flat no-lead (QFN) electrical component, reflow soldering was performed using a heated plate following the approximate reflow profile for NXP’s QFN components [37].

The FXTH87 sensors were flashed using demonstration code and were set to transmit continuously at a rate of 25 Hz (confirmed through oscilloscope tests). An FRDMKW019032 (FRDM) NXP transceiver (NXP Semiconductors) was used to receive the RF messages from the FXTH87 sensor. The FRDM transceiver sent the information via serial communication to a graphical user interface (GUI) provided by the vendor. 

The GUI displayed outputs including pressure, temperature, acceleration, voltage, and various status bits. The transducer data was recorded with 9-bit precision (512 analog-to-digital counts). Given that the FXTH87 sensor is being used to quantify deformation, the discretized data was recorded in arbitrary counts, as opposed to the manufacturer’s default setting of pressure in kPa. The temperature sensor has a reported precision of one degree Celsius over a range from −54 °C to 199 °C, however, the accuracy of the temperature sensor is not investigated in this paper. The FXTH87 incorporates dual-axis accelerometers; the Z-axis accelerometer is valid over a range of −30 *g* to 30 *g* whereas the X-axis accelerometer has a range of −10 *g* to 10 *g*. The manufacturer’s specified precision of the X and Z-axis accelerometers are approximately 0.039 *g* and 0.118 *g*, respectively.

### 2.3. Modifications to FXTH87 for Deformation Sensing

To transform the FXTH87 sensor from an ambient pressure sensor to a device that could measure load, an elastic coupling medium was required that would allow a mechanical indenter to depress the capacitive transducer. Without the introduction of a suitable coupling medium, the transducer would be easily damaged by the indenter. The ideal coupling medium would be an electrically insulative, two-part elastomer with a relatively low elastic modulus. In addition, said elastomer should be non-exothermic and room-temperature vulcanized. 

**Figure 3:**
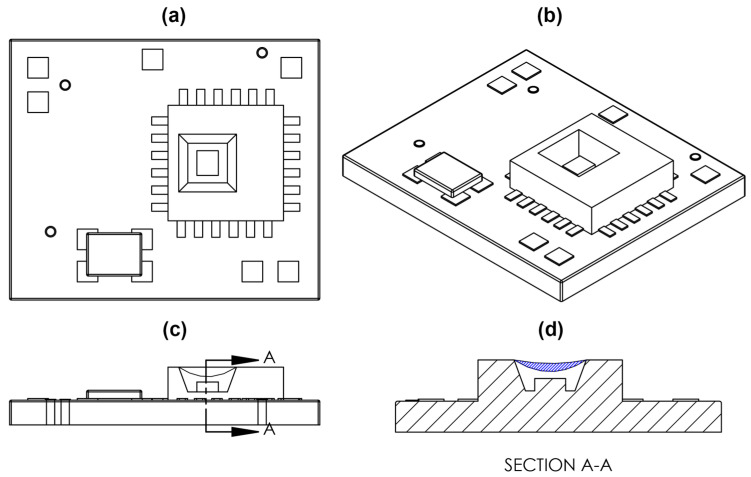
Sketch of FXTH87 on custom PCB. (**a**) Top view of FXTH87 and PCB. (**b**) Isometric view of FXTH87 and PCB. (**c**) Side view of FXTH87 and PCB. The Capacitive transducer membrane is defined by the concave line above the rectangular box, which depicts the location of the capacitive transducer. (**d**) Cross section view of the FXTH87 and PCB. Blue hatched region depicts the volume filled with Sylgard 184 on the surface of the capacitive transducer membrane.

The material chosen for the coupling medium was a commercial polydimethylsiloxane (PDMS) elastomer, Sylgard 184 (The Dow Chemical Company, Midland, MI, USA). The elastomer was applied to the external membrane of the capacitive transducer (Figure 3d) on ten FXTH87 sensors using a micropipette. The two-component Sylgard 184 composition followed the recommended 10 to 1 mix ratio [38]. Prior to the application of Sylgard 184 to the sensor package, the mixture was deaerated in a vacuum chamber at 50 kPa for 20 min. All ten sensors were laid flat and cured for a minimum of 48 h at room temperature before testing. 

The sensors were divided into two groups, based on the amount of Sylgard 184 applied to the external transducer membrane. The required volume of Sylgard 184 was predetermined from a micro-CT scan of the FXTH87; a 3D volume-of-interest software utility was used and found the volume of the external sensor cavity to be approximately 3 μL. The nominal prescribed volume was dispensed using a micropipette, however, variations in the deposited volume were observed due to the viscosity of uncured Sylgard 184. The actual volume of elastomer dispensed was verified gravimetrically using the known density of Sylgard 184, which is reported as 1.027 g/mL [38]. The sensors were then grouped based on the actual volume of dispensed elastomer. Group 1 and group 2 consisted of sensors with approximately 2 μL and 3 μL of Sylgard 184, respectively (Table 1). It is important to note that both groups had a visibly inward concave meniscus. Each group was tested to observe the effects of the elastomer volume on the maximum amount of compressive deformation that the sensor could withstand.

### 2.4. Calibration of Deformation and Load

**Figure 4:**
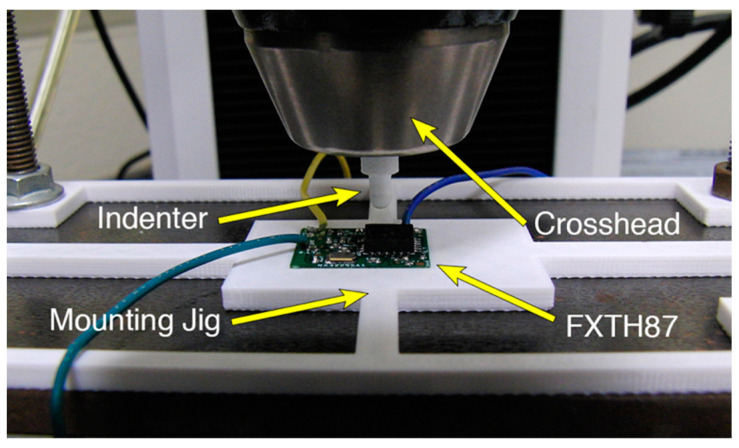
Instron 3343 applying compressive deformation to the FXTH87.

To calibrate the modified sensor package against both deformation and load, two experiments were performed. In the first case, a known value of surface deformation (i.e., indentation) was applied over the measuring range of the sensor. Compressive deformation was applied to the elastomeric coupling medium, using a commercial material testing machine (Model 3343, Instron, Norwood, MA, USA) and a hemispherical-tipped indenter. The indenter was 3D-printed in polylactic acid (PLA) plastic, with a tip radius of 1.6 mm. The sensor package was supplied with 3V using a voltage supply to eliminate the need for a battery and to ensure that the voltage level remained consistent between sensors and trials. The PCB was placed directly on the non-deformable metal plate at the base of the Instron, constrained within a 3D printed mounting jig that was used to align all of the sensors directly below the indenter (Figure 4). The mounting jig was also clamped to the plate to eliminate any movement. Compressive load was recorded using a 50 N load cell (Model 2519-102, Instron) and the Instron Bluehill software (Instron). Compressive load and deformation were recorded at a rate of approximately 14 Hz. Two sets of experiments were carried out based on the amount of Sylgard 184 adhered to the external transducer membrane. The maximum compressive deformation applied to the sensors with 2 μL and 3 μL of Sylgard 184 was 350 μm and 400 µm, respectively. Compressive deformation was applied at a rate of 350 µm/min for sensors with 2 μL of Sylgard 184 and 400 µm/min for sensors with 3 μL of Sylgard 184, to achieve full compressive deformation of each group of sensors in the same time interval.

**Figure 5:**
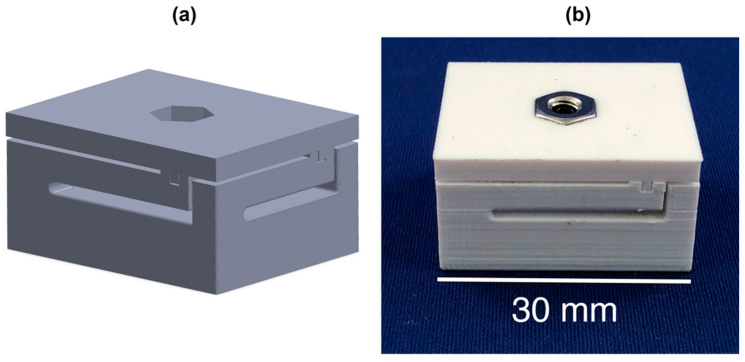
(**a**) SolidWorks model of compression enclosure. (**b**) 3D printed compression enclosure.

The aim of the second experiment was to calibrate a custom structural enclosure that was developed to demonstrate a proof-of-principle application of our sensor package (Figure 5). In other words, a custom load cell was developed and tested by combining a deformable structural component and a displacement sensor. While this enclosure is not an orthopaedic component, it is important to determine if the sensor is capable of being calibrated to measure loads in a simple deformable body. Successful calibration would indicate that the sensor is feasible to be embedded and calibrated within complex deformable bodies, such as custom orthopaedic components. The proof-of-principle deformable enclosure featured four cantilever beams designed to deflect approximately 500 µm under a compressive force. The lid of the enclosure was designed to hold a locknut and ball-point set screw with a ball radius of 1.25 mm. When a force is applied to the lid component the cantilever beams deform and the lid moves vertically downward towards the sensor. The locknut and ball-point set screw interact with capacitive transducer sensor as the enclosure deforms. The set screw acted as an adjustable indenter that allowed the starting point of the deformation to be altered. The enclosure was 3D printed in PLA using a Dremel 3D40 system (Dremel, Mt. Prospect, IL, USA); printing parameters were set to 100% infill density.

Calibration of the sensor package when it was embedded inside the compression enclosure was performed by applying compressive deformation to the enclosure and measuring the change in signal value and compressive load. The sensor package was held firmly inside the enclosure with wires protruding out of the base to an external power source, a DC power supply. The set screw housed in the lid was adjusted to a position that just barely came into contact with the surface of the capacitive transducer. This ensured that any amount of compression of the enclosure would result in displacement of the top plate of the capacitive transducer and increase the output signal value. Ten trials of compressive deformation were applied to the enclosure using an Instron 3343 and the flat head of a bolt measuring 1.5 cm in diameter.

A hysteresis test was performed on the compression enclosure and sensor package to determine the difference in signal output during loading and unloading cycles. During the loading phase, compressive deformation was applied to the enclosure and embedded sensor until the sensor output signal value reached its maximum capacity. The unloading phase returned the crosshead of the Instron to its original position at the start of the test. Five cycles of loading and unloading were applied to the package.

### 2.5. Acceleration Experiments

**Figure 6:**
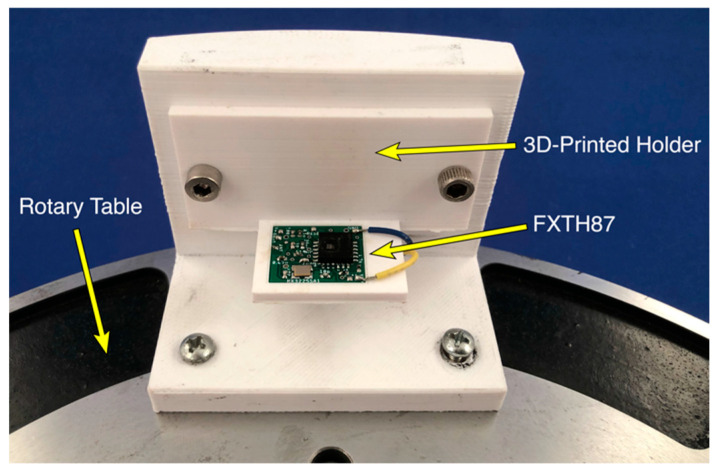
FXTH87 oriented to measure positive X-axis acceleration on rotary table.

To perform an acceleration calibration, each sensor package was secured in a custom 3D printed enclosure that was mounted to a rotary table (Model PSR300, Intellidrives, Philadelphia, PA, USA) (Figure 6). The sensor package was oriented so that the onboard X and Z axis accelerometers were in line with the rotational acceleration axis of the table. Note that the orientation of the package could be reversed within each enclosure to measure both positive and negative acceleration. The sensor package was powered using a 3V, 120 mA·h CR1632 coin battery to eliminate the need for wires connecting to a voltage supply. Constant rotational speed was used to apply rotational acceleration to the sensor package over the range of −5 *g* to +5 *g* at intervals of 0.5 *g*. The rotary table was allowed several seconds to achieve steady state prior to each increase in acceleration. The average and standard deviation of the experimental acceleration were obtained after reaching steady state at each acceleration level.

### 2.6. Power Management

The power consumption of the FXTH87 sensor was investigated to characterize the current draw and evaluate its feasibility for use in wireless telemetric orthopaedic implants. For this experiment, the FXTH87 was programmed to transmit at 1 Hz and the transmission power was set to the nominal value of 5 dBm. The input power trace of the FXTH87 was connected in series with a precision adapter, the µCurrent, and an oscilloscope was attached in parallel to the output terminals of the µCurrent adapter (EEVBlog, Sydney, Australia). The µCurrent adapter is a device that converts an input current to an amplified voltage for easier analysis. This device overcomes burden voltage, which is ideal since the FXTH87 circuit functions off a low voltage and minimal current draw. To achieve a clear waveform on the oscilloscope, the middle conversion factor of 1 mV/1 µA was selected on the µCurrent, however, a 1.2 Ω shunt resistor was placed in parallel between the input terminals of the µCurrent. The resulting effect was a conversion factor of 1 mV/10 µA from the µCurrent adapter to the oscilloscope.

### 2.7. RF Transmission

The ability to wirelessly transmit the sensor data from an implanted state is an important aspect of a telemetry system. To characterize the performance of the RF transmission of the FXTH87 in our proposed configuration, several tests were conducted to determine the maximum range of transmission. A baseline test was conducted in open-air, followed by tests in which the sensor was sandwiched between two layers of bovine muscle tissue; tests were repeated with three different tissue thicknesses on each side of the sensor (i.e., 17.5, 35 and 52.5 mm). Three trials were performed for each condition, and the maximum observed transmission distance was averaged.

### 2.8. Data Analysis

To analyze the data, resampling was required to achieve a consistent number of data points between the recorded data from the Instron and the FXTH87 sensor package. A MATLAB script was created to import the data sets for each trial into MATLAB (MathWorks, Natick, MA, USA). The recorded data from the sensor package was resampled using the built in MATLAB function, resample. The resulting sampling rate of the transducer matched the rate of the Instron.

To determine if there was a relationship between reported signal and compressive deformation, the output signal value from the FXTH87 sensor package was plotted against compressive deformation in Prism (GraphPad, San Diego, CA, USA). Separate graphs where generated to illustrate the performance of an individual sensor and the two groups of sensors filled with different amounts of Sylgard 184. A trendline was applied to the signal versus compressive deformation graph of sensor A over a linear region of the plot ranging from 150 to 350 µm to evaluate the sensitivity. The standard deviation of the signal units over the linear portion of this graph were averaged to determine the uncertainty in indenter position when the sensor package is displaying a specific signal value. Compressive load was plotted against compressive deformation to characterize the load required to reach specific levels of displacement and the maximum internal sensor resistive force. To develop a calibration curve for the compression enclosure, ten compression trials were performed; compressive load from the Instron was plotted against output signal value from the FXTH87 sensor package. The data was fitted to a non-linear Equation (1) generated by Prism:

(1) y(x)=Ax/(B+x)+Cx+D,

Using an additional compression trial, the measurement error of the load sensor was determined. To determine if the deformable enclosure and sensor exhibited hysteresis, the signal values from the sensor were plotted against load from the Instron 3343 during the cyclic deformation test.

For each of the ten sensors tested on the rotary table, the sensor data points were averaged at each acceleration level. To verify the expected linear correlation between the rotary table acceleration and the sensor acceleration, the sensor data for each board was shifted by a constant offset equal to its average sensor data with known zero acceleration. This alteration does not affect the trend in the sensor acceleration data but ensures that each sensor is calibrated to have approximately zero offset. To characterize the performance of the accelerometers, the average value and standard deviation of the experimental sensor data at each acceleration interval were plotted against the theoretical acceleration value. A linear trendline was applied to the data to generate a calibration curve for the X and Z-axis accelerometers.

The data points which were captured from the oscilloscope relating to the FXTH87 power consumption over an entire cycle were formatted using Excel software. The raw voltage data was converted to the corresponding current value using the 1 mV/10 uA conversion factor of the µCurrent adapter. The current waveform was broken up into three general regions: STOP1 (lowest power standby mode), sensor readings, and transmission. The process of sensor readings includes full sensor measurements and compensation. A noisy signal captured from the oscilloscope was subtracted off and then the average current draw for each mode was calculated using Excel. The area under the curve was used to determine the total milliamp-hours required for a 1 Hz transmission cycle. A graph displaying the FXTH87 sensor package’s instantaneous current draw was plotted against time.

## 3. Results

### 3.1. Compressive Deformation Results

**Figure 7:**
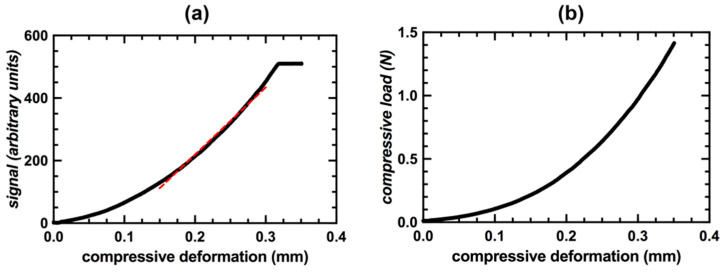
(**a**) FXTH87 signal value during compressive deformation for Sensor A. (**b**) Internal resistive force of Sensor A during compressive deformation.

The deformation experiments from the Instron demonstrated that there was a monotonically increasing relationship between the FXTH87 output signal value and compressive deformation. The plots for sensor A were highlighted to illustrate the performance of an individual sensor. The sensitivity of the signal versus compressive deformation plot for sensor A was 2.15 signal units/µm over the linear region of the graph (Figure 7a). The average standard deviation of the signal value over the linear region of the graph was 2.91 signal units. This corresponds to an uncertainty in position of ±1.35 µm. The inherent internal resistive force of the modified load sensor did not exceed 1.41 ± 0.01 N (mean ± standard deviation) (Figure 7b).

**Figure 8:**
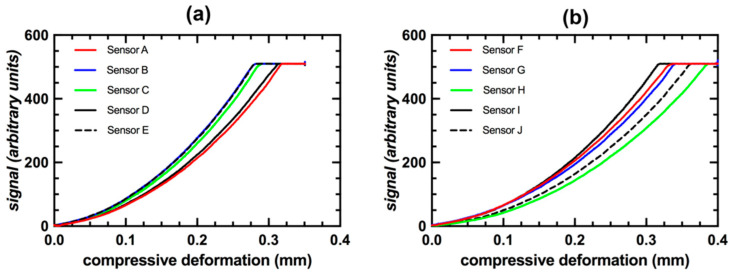
(**a**) FXTH signal value during compressive deformation for sensors in Group 1 (A–E). (**b**) FXTH signal value during compressive deformation for sensors in Group 2 (F–J).

The amount of compressive deformation and load required to reach the maximum output signal value was similar between sensors but never identical, even for sensors with the same amount of Sylgard 184. For the five sensors filled with 2 μL of Sylgard 184 it took 290 ± 16 µm of compressive deformation to reach the maximum output signal value (Figure 8a). The five sensors filled with 3 μL of Sylgard 184 took 350 ± 24 µm of compressive deformation to reach the maximum output signal value (Figure 8b). The maximum internal sensor resistive force required to cause complete compression of the capacitive transducer was 1.10 ± 0.07 N for sensors filled with 2 μL of Sylgard 184 and 1.19 ± 0.09 N for sensors filled with 3 μL of Sylgard 184.

**Figure 9:**
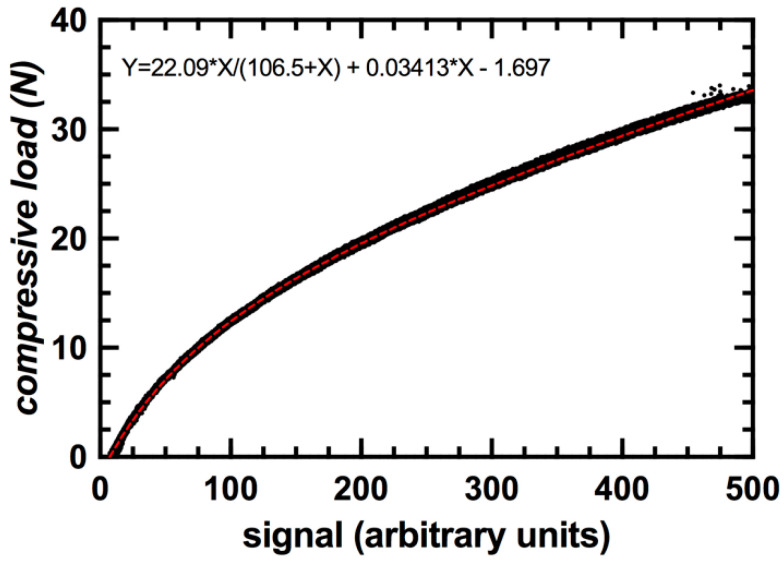
Calibration curve for sensor integrated within a 3D printed compression enclosure.

**Figure 10:**
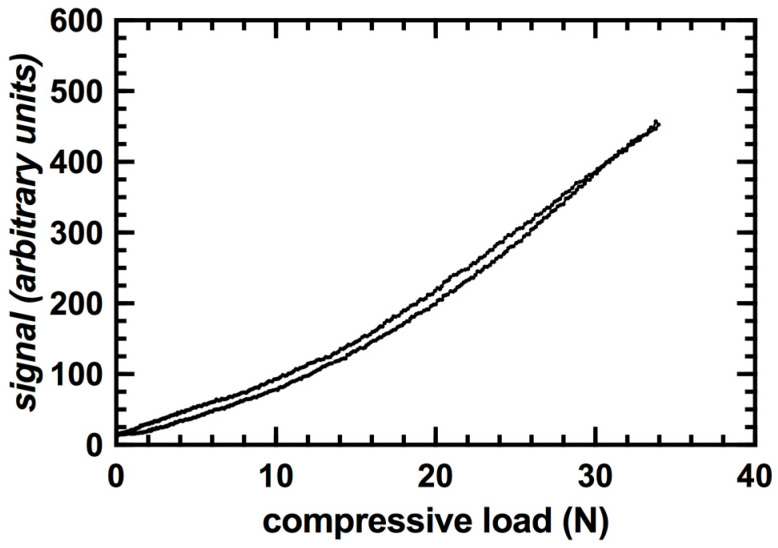
Hysteresis curve for sensor integrated within a 3D printed compression enclosure.

The compression enclosure exhibited a positive relationship between compressive load and signal value with an R^2^ value of 0.9992 (Figure 9). The average measuring error of the load sensor was less than 1%. A compressive load of approximately 35 N was required to fully compress the enclosure and the capacitive transducer. Hysteresis of the signal values corresponded to an average of less than 1 N between loading and unloading cycles (Figure 10). This value was consistent throughout all trials.

### 3.2. Acceleration and Power Management

**Figure 11:**
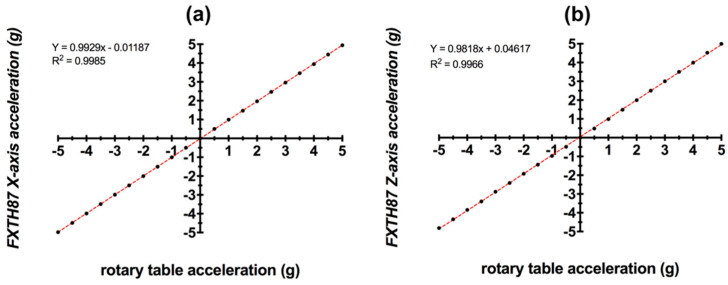
(**a**) Calibration curve for X-axis accelerometer for Sensors A–J. (**b**) Calibration curve for Z-axis accelerometer for Sensors A–J.

The R^2^ value of the linear regression analysis for the X and Z-axis acceleration was 0.9985 and 0.9966, respectively (Figure 11). The average standard deviation of the acceleration values for all the intervals combined was ±0.10 *g* for X-axis acceleration and ±0.15 *g* for Z-axis acceleration.

The average current draw of the FXTH87 while operating in standby was 14 µA, with a standard deviation of 115 µA. The average current draw while the sensor package is performing full measurements and compensations is 1.5 mA, with a standard deviation of 0.8 mA.

**Figure 12:**
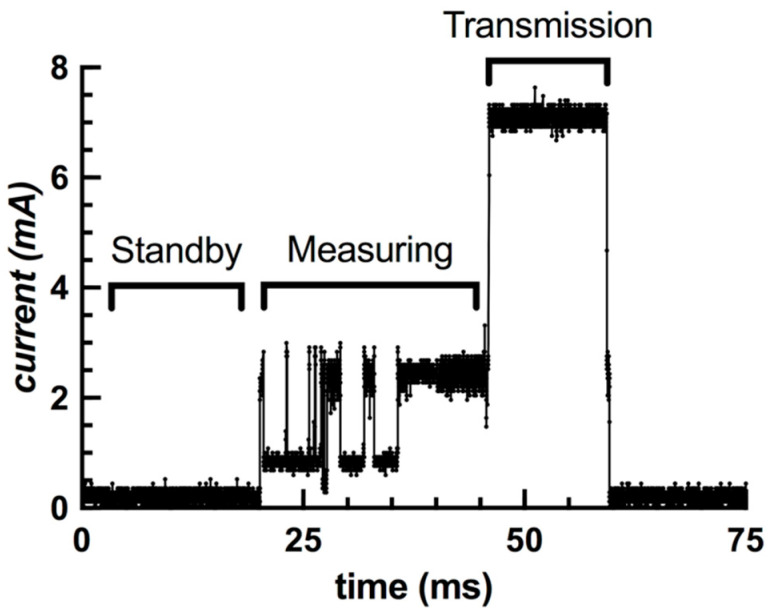
Current draw of the FXTH87 sensor package during a single transmission cycle.

The highest power consumption was observed while the sensor package was transmitting RF signals and was found to have an average current draw of 6.8 mA at 3.3 V, with a standard deviation of 0.7 mA (Figure 12). The total milliamp-hours required for one complete pulse width cycle was 4.09 × 10^−5^ mA·h.

### 3.3. RF Transmission

The maximum transmission distance in open air before the transceiver lost communication with the sensor was 9.5 m. Adding 17.5 mm of tissue had no discernable effect on the maximum transmission distance. Increasing the tissue depth to 35 mm and 52.5 mm caused the transmission distance to drop to 6 m and 4.5 m, respectively.

## 4. Discussion

We have demonstrated that it is feasible to modify a commercial wireless MEMS pressure sensor package to measure compressive deformation. Our investigation shows that there is a positive monotonic relationship between output signal value and the amount of compressive deformation applied. The FXTH87 tire pressure sensor package could detect very small changes in the deformation of its elastomer-coated diaphragm, allowing us to determine the position of an indenter to within ±1.35 µm. The maximum amount of compressive deformation that could be applied to the sensor ranged from 250 µm to 400 µm, depending on the amount of Sylgard 184 applied to the sensor diaphragm. This information is important because in future applications—where the sensor package is embedded—the enclosure must not exceed these deformation levels or else permanent damage may occur.

It is important to note that sensitivity and position uncertainty was determined over the region of the linear regression line (150 µm to 300 µm). The sensitivity and position uncertainty will change based on where the linear regression line is placed, however, this demonstrates that the sensor could be preloaded and operate in a linear region. This may be useful in certain applications where the required deformation is a fraction of the full range and a linear relationship is required.

The amount of Sylgard 184 applied to the sensor package had an influence on the outcome. Sensors that were filled with more Sylgard 184 required more compressive deformation to reach the maximum signal value. This was expected since the mechanical indenter had to compress slightly more Sylgard 184 during deformation. With this information, a larger volume of Sylgard 184 could be applied to the pressure transducer to increase the amount of compressive deformation that the FXTH87 package could measure. On the contrary, the smaller volume of Sylgard 184 would be beneficial in applications such as orthopaedic sensing, as the full range of signal values can be obtained with minimal deformation. Due to the size restrictions of many implants, it is expected that an implant capable of deforming onto the sensor would only be deforming a very small amount. If deformation of the implant was larger than the capacity of the sensor, the mechanism contacting the capacitive transducer could be adjusted to prevent sensor overloading. The internal sensor resistive force was similar within each set of sensors, likely due to the small discrepancy in the amounts of Sylgard 184. It would be expected that a larger quantity of Sylgard 184 would increase the compressive load required to cause full deformation of the transducer. Although compressive deformation was applied at a linear rate, both the applied load and thus signal value were not linearly correlated with the compressive deformation. When Sylgard 184 is under compression, the stress/strain curve has a linear elastic region until strain values of approximately 55%, which is then followed by a nonlinear region [39]. During the tests performed in this study, it is expected that the Sylgard 184 remains in the linear elastic region of the stress/strain curve as two distinct phases are not observed in the data. The characteristics of the capacitive transducer are likely responsible for the observed non-linearity, as capacitance is inversely proportional to the distance between the parallel plates of the transducer (C ∝ 1/d). Even though the signal value is not linearly correlated with deformation, within each sensor package the relationship is consistent and repeatable every time compressive deformation is applied. This repeatability allows a calibration curve to be generated for a compression enclosure with an embedded FXTH87 sensor package. The FXTH87 package was also calibrated to act as a load sensor by quantifying the relationship between the signal value and the compressive load. Once the calibration was generated, the compressive load can be directly determined by monitoring the output signal value from the FXTH87. The compact wireless telemetric sensor proposed in this study was effectively tasked to measure micrometer-level deformation and load in a small-scale compression enclosure.

Using the calibration curve that was generated for our 3D printed strain member, the FXTH87 could be used to measure both small-scale deformation and loads of up to 35 N. The small internal resistive force of the FXTH87 is inherently accounted for during the calibration of the deformable member. The error of the load sensor is low; however, it could be further reduced by performing additional calibration trials, therefore improving the quality of the calibration curve. The sensor package is capable of being embedded within enclosures of all sizes and fabricated from any material; thus, there is no limit on the amount of load the package can measure, as long as the deformation resulting from that load is within the measurable range of 250–400 µm. This principle is utilized in many commercial load sensors with varying capacities; the same strain gauge can be used to record measurements, but the structure of the strain member is altered to change the operating range. The hysteresis exhibited between the loading and unloading cycles of the compression enclosure was a small and consistent value throughout all tests. This will allow a future user to easily correct for the small discrepancy between cycles during calibration experiments. It is expected that a majority of the observed hysteresis is a result of the plastic compression enclosure, and not due to the sensor and the applied Sylgard 184. Pure Sylgard 184 has been shown to have minor hysteresis [40]. In future applications, if minimizing hysteresis is a primary goal, it is important to embed the sensor in an enclosure with minimal inherent hysteresis.

This study has shown that there is a positive linear relationship between experimental and applied acceleration, with a constant offset that could be compensated through calibration. This result was important to obtain, as this information shows that this package is capable of measuring acceleration along two axes in enclosures of all sizes. Calibrating the accelerometers allows the possibility of using the accelerometers as a trigger to alter the measurement duty cycle of the sensor package. This scenario would be likely in the case where batteries are tasked to power the package. The accelerometers could be used to temporarily increase the duty cycle during periods of higher acceleration and then lower the duty cycle during rest. Carefully controlling the number deformation/load measurements and RF transmissions will assist in prolonging the lifetime of the battery.

The experiments relating to the power consumption of the FXTH87 were very promising, indicating the feasibility of using a coin-cell battery to power the sensor package inside orthopaedic implants. The results showed that the FXTH87 consumed about 14 ± 115 µA while operating in the lowest power standby mode. Based on the high standard deviation, this measured current usage is primarily dominated by noise, despite the attempt to correct for noise. The FXTH87 data sheet states that the current consumption in STOP1 should be about 1 µA, which suggests that power consumption could be even lower than reported here. The milliamp-hours required for a one second cycle of standby, sensor acquisitions, and transmission in the FXTH87 was found to be 4.09 × 10^−5^ mA·h, whereas a typical CR-1632 battery provides capacity of approximately 120 mA·h. The FXTH87 could therefore operate for about 2.9 million transmission cycles. When acquiring and transmitting at the maximum rate of 25 Hz, the average power consumption of the FXTH87 sensor package can be as low as 11 mW; in this configuration, the device would operate for over 36 h from a 120 mA·h battery. During standby mode, the FXTH87 was measured to have a power consumption of 42 µW, meaning it would last at least one year with the same power source. In a scenario where the FXTH87 is embedded in an implant, it is highly unlikely that the maximum transmission rate would be required at all times; typically, the FXTH87 would be programmed to trigger transmission at its maximum capacity of 25 Hz for a brief window, lasting no more than a few minutes. This would allow a clinician to monitor the patient data during an examination, then the FXTH87 would return to standby mode to prolong the battery life of the package. In other words, the device will almost always be operating in one of two states, standby mode or at maximum transmission capacity. This is beneficial in cases where orthopaedic implants need to be monitored at several timepoints in the weeks or months following surgery. In comparison, the power consumption of the 9-channel telemetry system described in [28] is approximately 5 mW when transmitting at a rate of 125 Hz. While the power consumption of the telemetry system used to measure intra-articular tibial forces was not specified, it was stated that 40 mW was adequate to power the system [11]. The sensor package and telemetry system devised in [15] required approximately 230 mW during transmission, 1.7 mW during sensor measurements, and 320 µW during standby. The FXTH87 is comparable to the previously described telemetry packages in terms of power consumption, and thus should be feasible for use in orthopaedic applications.

Based on the RF transmission tests conducted in this paper, it is clear that the sensor package in its described configuration is capable of transmitting signals through tissue. These results are significant as it proves that a traditional antenna component is not required in this design, allowing for the size of the sensor PCB to be minimized. Noting that transmission distance decreases with increased tissue depth, it is possible that the default 5 dBm output power of the FXTH87 might need to be increased in some applications. The FXTH87 transmission output power can be adjusted through the Dynamic RF Power Correction firmware routine [33].

While the objective of the FXTH87 sensor package is similar to previously developed embedded systems, the method of measuring deformation and load described in this study presents a number of unique advantages. The FXTH87 sensor and circuit board is effectively able to record and transmit sensor data in a package that occupies a volume less than a cubic centimeter. The full extent of the size of the implantable 9-channel telemetry system and strain gauges developed in [28] is never explicitly stated. However, it is evident that the volume of this package is significantly greater than the FXTH87 package, as the telemetry system occupies a large portion of the head and stem of joint replacement implants. Similar telemetry systems developed by [11,14], face the same challenges, as the requirements for inductive coupling occupy a majority of the internal cavity of the implants. While some very small strain monitoring systems have been developed for intramedullary nails [21,22], they have not been successful due to the poor signal strength of their respective telemetry systems and a lack of supporting strain gauges. The FXTH87 sensor package has no practical limitations on the amount of load that it can measure, as long as the deformation resulting from the applied force does not exceed the maximum deformation capability of the capacitive transducer. In the current study, tests with a compression enclosure containing the capacitive transducer resulted in a load measurement error of less than 1%, which is comparable with previously described sensor packages. For example, a hip prosthesis developed by [6] that utilizes the telemetry system described in [28] was calibrated to measure forces ranging from 2 to 5 kN, with an average measuring error ranging from 0.4% to 0.9%. An instrumented tibial implant developed in [11] was calibrated to measure loads of 2000 N with average errors of around 1%.

In terms of size comparison, the three telemetry systems described in [11,15,28] all occupy a significantly larger volume than the FXTH87 package due to the transmitting circuitry and additional components required for inductive coupling. The FXTH87 is competitive in terms of low-power consumption in comparison to other devices but it is limited to transmission rates less than 25 Hz. While the FXTH87 sensor package is one of the smallest sensing packages available, it also provides more supporting components than the previously described sensing and telemetry devices. In addition to the capacitive transducer, RF transmitter and 8-bit 4 MHz central processing unit (CPU) the FXTH87 also features a temperature sensor, two axis accelerometer, 125 kHz LF receiver, internal timers and clocks, 6 channel 10-bit ADC, 16 KB of flash memory, and 512-byte RAM. The miniature size of the FXTH87 package along with the built-in components make this sensor package an effective solution to record and transmit load, deformation, temperature, and acceleration.

One potential limitation of this approach is the possibility of hysteresis and stress relaxation, which might alter the calibration over time. The elastomer used here (Sylgard 184) is a viscoelastic material and exhibits properties such as creep and stress relaxation [41,42]. It is expected that in certain applications of the FXTH87 sensor package, an indenter may be holding the coated capacitive transducer membrane in a compressed state for extended periods of time. When held in compression at a specific strain value, the load and signal value are expected to decrease slightly over time, due to stress relaxation of the PDMS elastomer. The experiments presented in this study were performed immediately after the elastomer had cured. In future experiments, the sensors should be preconditioned, which would allow the elastomer to undergo compression set prior to calibration, therefore, reducing the effect of stress relaxation and drift of the signal value over extended periods of compression. This phenomenon does not influence the outcome of this study; however, it may alter the profile of the calibration curve. Depending on the application, the sensor package could be exposed to large amounts of cyclic loading. While the tests performed in this study demonstrate small hysteresis in the short term, a long-term hysteresis experiment should be performed to quantify the FXTH87 signal difference between the loading and unloading cycles. The mixture ratio for the two components of the elastomer used in this study was based on the Dow Chemical Company published guidelines for Sylgard 184 preparation [38]. It is expected that the mechanical properties of the Sylgard 184 could be altered by changing the mixture ratio of the base and curing agents. Altering the viscoelastic properties of the transduction medium could further minimize stress relaxation and hysteresis. Alternatively, another type of elastomer could have been used to achieve similar functionality to the Sylgard 184. It was beyond the scope of this study to evaluate alternative elastomers and Sylgard 184 compositions. While other options could be explored, the performance of the Sylgard 184 was sufficient for the intended future application.

The target application of the FXTH87 wireless telemetric load and deformation sensor is orthopaedic implants that are too small to accommodate conventional strain-gauge sensing packages. The results of this study show that a repurposed tire-pressure sensor may be feasible for use in orthopaedic applications, as it is capable of monitoring small-scale deformation that can be calibrated into load measurements, has minimal power consumption, and can effectively transmit through tissue. Depending on the accompanying spring body, the sensor could be used in regenerative medicine and tissue engineering applications [43]. For example, the proposed package could potentially be embedded within fracture fixation plates to monitor the load and strain acting on the plate during patient rehabilitation. This information could be used to predict failure of the fixation plate and monitoring the plate strain over time should quantify the process of bone union. In addition, in the future it may be possible to connect supporting sensors to unused analog-to-digital converters on the FXTH87 to monitor physiological variables such as pH, oxygen tension, and other biomarkers [43]. Current mechanisms of measuring load in fracture fixation plates depend on sensors monitoring the change in surface strain on the implant. It may be possible to convert this flexure force into a compressive force, which would allow the proposed sensor to be embedded within a fixation plate to record load data. The proposed sensor inherently measures a vertical force; however, it is possible to embed the sensor in custom enclosures that transduce flexure, tension, and torque into compressive forces. In addition, the vertical mechanism of load measurement is well-suited for implants that are naturally under compression. For example, the sensor could also be embedded within intervertebral spinal-fusion cages to monitor in-vivo spinal loads and the bone healing process following spinal-fusion surgery. Another example application could be the measurement of loads inside a high-tibial osteotomy implant following surgery. The FXTH87 sensor package could also replace the sensor packages currently used to measure loads in larger components, such as knee implants. The information that could be obtained from these instrumented implants could improve future implant design and assist physicians in guiding patient rehabilitation practices. Knowledge of the forces acting on implants could also allow patients to identify at risk activities and self-monitor their recovery process. While the proposed sensor package is a key feature of a telemetric implant, there are several other factors that must be taken into consideration to ensure that the package is successful. For instance, the package must be sealed in a hermetic enclosure to prevent harm to the patient. The RF signal from the device must be capable of passing through said hermetic enclosure to an external receiver. Finally, the device, power source, and supporting implant must adhere to regulatory guidelines before being safely implanted in a patient. Outside of orthopaedic applications, this sensor package has the capability of being utilized for many load and strain sensing applications due to its size and functionality. These applications include, but are not limited to, in-vivo pressure measurements, activity tracking, infection monitoring (via temperature), and benchtop research experiments using cadaveric specimens. In the current enclosure configuration, the sensor package was designed to measure compressive deformation and loads; however, this sensor could also be embedded within custom structural enclosures that are designed to transduce tension, flexion, and torque.

## 5. Conclusions

We have demonstrated that a commercially available MEMS pressure sensor can be converted into an effective tool to measure deformation and load. The device has integrated RF transmission and power management capabilities, facilitating its use as a low-power, miniature sensor package for orthopaedic applications. With the addition of an elastomeric coating to the external surface of the capacitive transducer, the sensor package is suited to measure changes in deformation over a range of 350 µm. The position of a mechanical indenter can be determined to within about 1 µm. The repeatability of the output signal profile during compressive deformation allowed the sensor package to be calibrated to measure load. The package can be tasked to measure a wide variety of load magnitudes by altering the compressive enclosure, as long as the amount of external deformation onto the sensor package does not exceed the maximum limits defined in this paper. The onboard accelerometer was calibrated and may be an effective tool to record implant motion during periods of activity. The low-power consumption of the sensor package allows it to pair with a battery or alternative power source to measure and transmit data over longer periods of time. In-vivo load and deformation information has been shown to be beneficial in the development of new orthopaedic implants and patient rehabilitation practices [1,2] by monitoring the forces acting on the implants and surrounding structures. The novel device described in this study has the potential to provide wireless real-time deformation and load information, while embedded within orthopaedic implants and other deformable strain members.

## Figures and Tables

**Table 1:** Volume of Sylgard 184 applied to FXTH87 sensors.

Sensor (Group 1)	Sylgard 184 Volume (μL)	Sensor (Group 2)	Sylgard 184 Volume (μL)
A	2.26	F	3.44
B	1.96	G	2.75
C	2.26	H	3.24
D	2.26	I	2.85
E	2.06	J	2.95
